# Silica Gel-Mediated Organic Reactions under Organic Solvent-Free Conditions

**DOI:** 10.3390/molecules171011469

**Published:** 2012-09-27

**Authors:** Satoaki Onitsuka, Yong Zhi Jin, Ajam C. Shaikh, Hiroshi Furuno, Junji Inanaga

**Affiliations:** Institute for Materials Chemistry and Engineering (IMCE), Kyushu University, Hakozaki, Higashi-ku, Fukuoka 812-8581, Japan

**Keywords:** aromatic nitration, green synthetic process, heterogeneous reaction, Morita-Baylis-Hillman reaction, one-pot Wittig-type olefination, solid reaction medium

## Abstract

Silica gel was found to be an excellent medium for some useful organic transformations under organic solvent-free conditions, such as (1) the Friedel-Crafts-type nitration of arenes using commercial aqueous 69% nitric acid alone at room temperature, (2) one-pot Wittig-type olefination of aldehydes with activated organic halides in the presence of tributyl- or triphenylphosphine and Hunig’s base, and (3) the Morita-Baylis-Hillman reaction of aldehydes with methyl acrylate. After the reactions, the desired products were easily obtained in good to excellent yields through simple manipulation.

## 1. Introduction

In the chemical industry, huge amounts of organic solvents have been used and are still wasted all over the World. The development of no-use or a large-scale cut in their use has, therefore, become an increasingly important issue of synthetic organic chemistry, from not only a practical but also an environmental point of view [1]. There have been several approaches to access to this problem, e.g., the developments of neat reactions that proceed under various conditions such as microwave irradiation, thermal heating, grinding, sonication, *etc*., or in organic or inorganic solid-media, or in ionic liquid-media under organic solvent-free reactions [2,3,4,5,6,7]. In this context, we have made our own efforts to contribute to this research field developing some useful synthetic methods that do not require any organic solvents as reaction media [8,9,10,11,12]. Silica gel has widely been utilized, not only as an effective adsorbent for chromatography, but also as a mild acid catalyst, an accelerator, or a reaction medium which is easily separable from the products after the reaction [7,13]. We report here the successful use of silica gel as a solid reaction medium for three synthetically useful organic transformations: aromatic nitration, Wittig-type olefination, and Morita-Baylis-Hillman reaction, in which no organic solvents are required.

## 2. Results and Discussion

### 2.1. Nitration of Aromatic Compounds with 69% Nitric Acid

Aromatic nitration is one of the most important and convenient methods to introduce nitro group(s) into aromatic nuclei, and a number of nitrating reagents and reaction conditions have so far been developed for this purpose [14,15]. So-called mixed acid (HNO_3_^conc.^ + H_2_SO_4_^conc.^) has been the most popular way to generate the nitronium ion (NO_2_^+^) as the active species for the Friedel-Crafts-type nitration since nitric acid is a good nitronium ion precursor in terms of cheapness, handleability and atom economy. However, the development of more convenient and practical methods that do not require such strong acids, acidic additives, or even organic solvents has been strongly desired from an environmental point of view [1], and a variety of green chemical approaches using nitric acid as a nitration reagent have been made in recent years [16,17,18,19,20,21,22,23,24,25,26,27,28,29]. Although aromatic nitration using nitric acid has long been known, there are few examples in which nitric acid was simply used as a nitrating agent under solvent-free conditions [26,27,28,29]. We report here the usefulness of silica gel as a solid reaction medium for the aromatic nitration using commercial 69% nitric acid at room temperature [30,31].

**Scheme 1:**
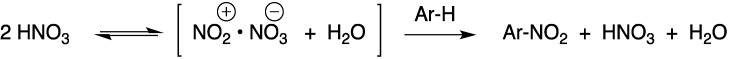
A scheme for the aromatic nitration using nitric acid in the absence of sulfuric acid.

Nitric acid can provide nitronium ion in equilibrium as shown in Scheme 1. Therefore, if one could develop an efficient dehydrating system for this process, concentration of the nitronium ion would be increased to react easily with aromatic compounds under mild and clean conditions leaving only water as a waste. Based on this idea, we tried to use silica gel as an absorbent for water and also as a dispersant for the substrates to achieve an activator-free and organic solvent-free nitration of non-activated and activated aromatic compounds.

In Table 1 the results of the silica gel-mediated nitration of ethylbenzene (1 mmol) with 69% HNO_3_ under various conditions are summarized. Although an excess amount of nitric acid was needed for completion of the reaction, the nitration proceeded smoothly at room temperature in 500 mg of silica gel to give the desired products in almost quantitative yield. The reaction was obviously accelerated by the addition of silica gel [COSMOSIL 75SL-II-PREP (Nacalai Tesque)] (entries 6 *vs.* 8). The use of another kind of silica gel, such as Silica gel 40 (0.2–0.5 mm, Merck), Silica gel 60 (0.2–0.5 mm, Merck), BW-300 (40 μm, Fuji Silysia), Silica gel 60 (40–50 μm, Kanto Chemical), and Silica gel 60 N (63–210 μm, Kanto Chemical), also gave a similar result, but other inorganic solids having dehydrating ability like molecular sieves 4Å and anhydrous MgSO_4_ were less effective for this transformation. The concentration of nitric acid is crucial; When 60% nitric acid was used in place of 69% nitric acid, the product yield significantly dropped (entries 7 *vs.* 11).

**Table 1:** The nitration of ethylbenzene with nitric acid on silica gel ^a^. 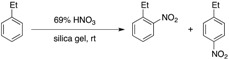

Entry	69% HNO_3_	Silica gel ^b^	Time	Yield ^c^	Ratio ^d^
	mmol	mg	h	%	*o*-/*p*-
1	1.1	250	1	16	42/58
2	1.1	250	12	24	43/57
3	2.0	250	1	26	43/57
4	4.0	250	1	45	43/57
5	6.0	500	1	70	43/57
6	8.0	500	1	81	44/56
7	8.0	500	12	97 (85)	44/56
8	8.0	none	1	60	46/54
9	8.0	none	12	89	47/53
10	1.1 (60% HNO_3_)	250	1	6	44/56
11	8.0 (60% HNO_3_)	500	12	8	44/56
12	8.0 (60% HNO_3_)	none	1	4	45/55

^a^ Ethylbenzene (1 mmol) was used; ^b^ COSMOSIL 75SL-II-PREP (Nacalai Tesque) was used; ^c^ ^1^NMR yield using pentamethylbenzene as an internal standard. Isolated yield is given in parenthesis; ^d^ Determined by ^1^H-NMR.

The nitration of naphthalene with 1.1 eq of 69% nitric acid at room temperature for 1 h afforded mononitronaphthalene in good yield as a mixture of regioisomers (α/β = 97:3) (Table 2). The yield gradually increased as the reaction time was increased (entries 1 *vs.* 2), and when naphthalene was treated with two equivalents of nitric acid for 12 h, the products were obtained almost quantitatively (entry 4). As far as this substrate is concerned, little effect of silica gel was observed: comparable results were yielded under neat conditions (entries 1 *vs.* 5 and 4 *vs.* 6). This may be attributed to the inefficient dispersion of naphthalene on silica gel because both of them are solids at room temperature. 

**Table 2:** The nitration of naphthalene with nitric acid on silica gel. 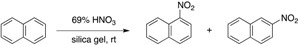

Entry	Naphthalene	69% HNO_3_	Silica gel ^a^	Time	Yield ^b^	Ratio ^c^
	mmol	mmol	mg	h	%	1-/2-
1	1.0	1.1	250	1	72	97/3
2	1.0	1.1	250	12	82	96/4
3	1.0	1.5	250	12	94	96/4
4	1.0	2.0	250	12	97 (94)	97/3
5	5.0	5.5	none	1	74	97/3
6	2.5	5.0	none	12	95	97/3

^a^ COSMOSIL 75SL-II-PREP; ^b^ ^1^NMR yield using pentamethylbenzene as an internal standard. Isolated yield is given in parenthesis; ^c^ Determined by ^1^H-NMR.

**Scheme 2:**
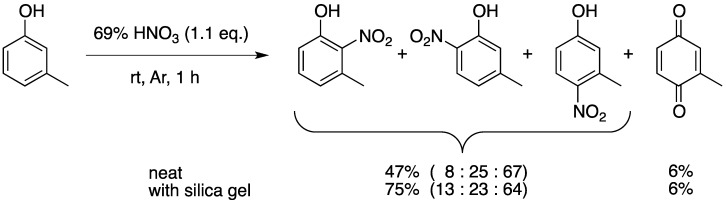
The nitration of *m*-cresol using 69% nitric acid with or without silica gel.

The nitration of *m*-cresol was also investigated. The high reactivity of this substrate tends to afford an oxidation product and tarry substances as byproducts. On the other hand, the use of silica gel with 1.1 eq of nitric acid largely improved the yield of the desired nitro compounds as shown in Scheme 2. The observed *para*-selectivity (64%–67%) appears to be slightly higher than the reported ones (40%–63%) obtained under other reaction conditions shown in Table 3. The nitration of phenols and related compounds using silica gel-supported nitric acid, which was prepared by treating silica gel with 8N nitric acid for 2 h followed by filtration and drying, has been reported [32]. In this case, however, the reaction was carried out in dichloromethane.

**Table 3:** Some reported examples of the nitration of *m*-cresol under various conditions.

Conditions	Yield (%) (2:6:4)	Reference
70% HNO_3_/H_2_SO_4_/0 °C (direct)	36 (24:25:51)	[33]
NaNO_3_/NaNO_2_/3M H_2_SO_4_/ether/rt	91 (25:30:40)	[34]
60% HNO_3_/Yb-Mo-HKSF/THF/rt	91 (14:29:57)	[23]
Fe(NO_3_)_3_/Clayfen/ether/rt	54 (~:37:63)	[35]

### 2.2. One-Pot Wittig-Type Olefination of Aldehydes

The Wittig reaction is one of the powerful tools to install a carbon-carbon double bond in a highly selective manner [36]. For the Wittig reaction using stabilized phosphonium ylides under solvent-free conditions, there have been various approaches, such as enantioselective reaction in chiral solid media [37], fusion of substrates under microwave irradiation [38,39], grinding of reagents [40,41,42], activated alumina promoted reaction [43], and rate acceleration by immediate solvent evaporation [44]. Although these methods are attractive and environmental friendly, some of the reactions needed special equipment like a microwave reactor or a ball-milling machine and produced unsatisfactory isolated yields of the products.

**Scheme 3:**
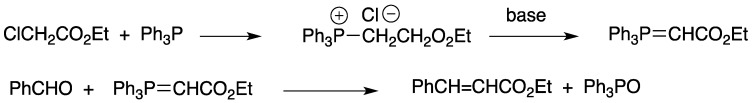
Whole process of a typical Wittig reaction using a stabilized phosphonium ylide.

We report here a quite convenient method for the direct one-pot Wittig-type olefination of aldehydes using ethyl chloroacetate, a phosphine, and a base where the following three processes would take place: the phosphonium salt-formation, the ylide-formation, and the Wittig reaction to give the α,β-unsaturated esters as the final products (Scheme 3) [31]. The fact that silica gel can accelerate the Wittig reaction of aldehydes with stabilized phosphonium ylides in organic solvent has been reported [45].

Taking benzaldehyde (1 mmol), ethyl chloroacetate (1 mmol), and triphenylphosphine (1 mmol), we first examined the effects of reaction medium and the base (Table 4). All the reactions were conducted at 90 °C by considering the melting point of triphenylphosphine. Among the reaction media tested, silica gel was found to be the best, and ethyl cinnamate was obtained almost quantitatively when diisopropylethylamine was employed as a base (entry 8). Neat conditions or the use of toluene, organic polymer or alumina as a reaction medium were not effective (entries 9–15). Interestingly, the use of a stronger base such as 1,8-diazabicyclo[5.4.0]undec-7-ene (DBU) or phosphazene gave rise to unexpectedly low yields (entry 6 and 7). In these cases, their high nucleophilicity and strong basicity seem to cause the ammonium salt formation and/or the Darzens reaction, though the formation of such byproducts has not been ascertained experimentally. Suitable basicity and relatively low nucleophilicity of diisopropylethylamine would be a key of the present success. Simple treatment of the whole mixture with a mixture of hexane-ethyl acetate (20:1) through a column followed by evaporation of the solvents afforded the olefination product in a practically pure state. 

**Table 4:** One-pot Wittig-type olefination of benzaldehyde under various conditions^ a^. 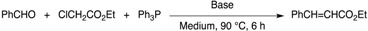

Entry	Medium	Base	Yield (%) ^b^	*E*/*Z* ^c^
1	silica gel	none	18	95/5
2	silica gel	Ph_3_P	32	95/5
3	silica gel	Na_2_CO_3_	29	92/8
4	silica gel	KOH	42	91/9
5	silica gel	Et_3_N	86	91/9
6	silica gel	DBU	25	89/11
7	silica gel	Phosphazene	20	93/7
8	silica gel	*i*-Pr_2_NEt	99	93/7
9	none	*i*-Pr_2_NEt	67	94/6
10	toluene	*i*-Pr_2_NEt	43	94/6
11	PTFE ^d^	*i*-Pr_2_NEt	56	93/7
12	PSDVB ^e^	*i*-Pr_2_NEt	58	92/8
13	alumina (acidic)	*i*-Pr_2_NEt	40	93/7
14	alumina (neutral)	*i*-Pr_2_NEt	36	92/8
15	alumina (basic)	*i*-Pr_2_NEt	41	94/6

^a^ Benzaldehyde (1 mmol), ethyl chloroacetate (1 mmol), triphenylphosphine (1 mmol), a base (1 mmol), and medium (1 g) were used; ^b^ GC yield using *n*-tetradecane as a standard; ^c^ Determined by GC; ^d^ PTFE: Polytetrafluoroethylene; ^e^ PSDVB: Poly(styrene-*co*-divinylbenzene).

The present silica gel-mediated one-pot olefination reaction was successfully applied to various aldehydes and ketones. As shown in Table 5, the reaction of aromatic aldehydes proceeded smoothly under the standard conditions to give the corresponding olefins in good to excellent yields (runs 2–6). On the other hand, the reaction of aliphatic aldehydes having acidic hydrogen atoms at the α-position of the carbonyl group produced a mixture of more than three olefination products as regio- (α,β-unsaturated and β,γ-unsaturated) and stereoisomers (*E* and *Z*) (entries 7 and 11). Moreover, in these cases, the aldol-type and other types of reactions that proceed through enolate formation became major. Such kind of side reactions, however, could be significantly suppressed by employing tributylphosphine at room temperature instead of triphenylphosphine at 90 °C (entries 8, 10, and 12). The observed superiority of tributylphosphine over triphenylphosphine suggests that the phosphonium salt-formation would be the rate-determining step of the present one-pot olefination.

**Table 5:** One-pot Wittig-type olefination of various aldehydes on silica gel ^a^. 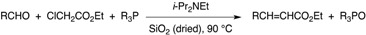

Entry	RCHO	R_3_P	Time (h)	Yield (%) ^b^	*E*/*Z* ^c^
1	PhCHO	*n*-Bu_3_P	2	99	95/5
2	4-MeOC_6_H_4_CHO	Ph_3_P	6	96	94/6
3	4-MeC_6_H_4_CHO	Ph_3_P	6	93	94/6
4	4-ClC_6_H_4_CHO	Ph_3_P	6	99	92/8
5	4-NCC_6_H_4_CHO	Ph_3_P	2	96	90/10
6	4-O_2_NC_6_H_4_CHO	Ph_3_P	2	83	90/10
7	*n*-C_6_H_13_CHO	Ph_3_P	6	11	85/15
8	*n*-C_6_H_13_CHO	*n*-Bu_3_P	24^ d^	54	96/4
9	*c*-C_6_H_13_CHO	Ph_3_P	6	78	97/3
10	*c*-C_6_H_13_CHO	*n*-Bu_3_P	24^ d^	99	98/2
11	PhCH_2_CH_2_CHO	Ph_3_P	6	11	87/13
12	PhCH_2_CH_2_CHO	*n*-Bu_3_P	6^ d^	46	97/3

^a^ An aldehyde (1 mmol), ethyl chloroacetate (1 mmol), a phosphine (1 mmol), diisopropylethylamine (1 mmol), and silica gel (1 g) were used; ^b^ GC yield; ^c^ Determined by GC; ^d ^The reaction was carried out at room temperature.

**Scheme 4:**
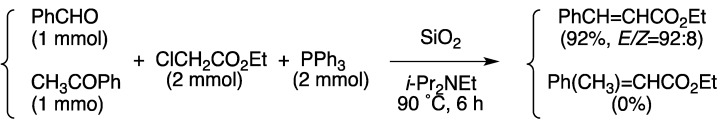
The competitive reaction of benzaldehyde *vs.* acetophenone.

Low reactivity of ketones enabled the highly chemoselective olefination of aldehydes in the presence of ketones. A typical example is shown in Scheme 4.

Table 6 shows the applicability of the present silica gel-mediated one-pot olefination of aldehydes to other organic halides, such as benzyl chloride, α-bromo-γ-butyrolactone, and phenacyl bromide. As expected, the corresponding olefination products were conveniently obtained in good isolated yields.

**Table 6:** One-pot olefination of benzaldehyde with various halides on silica gel ^a^. 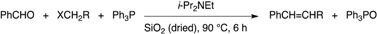

Entry	X, R	Yield (%) ^b^	*E*/*Z* ^c^
1	Cl, Ph	66	67/33
2	α-Br-γ-butyrolactone	52	95/5
3	Br, COPh	85	>99/1

^a^ Benzaldehyde (1 mmol), a halide (1 mmol), triphenylphosphine (1 mmol), diisopropylethylamine (1 mmol), and silica gel 40 (0.2–0.5 mm, Merck, 1 g) were used; ^b^ Isolated yield; ^c^ Determined by GC.

### 2.3. The Morita-Baylis-Hillman Reaction

The Morita-Baylis-Hillman (MBH) reaction possesses the two most important requirements: atom economy and generation of multi-functional groups, and, therefore, it has attracted many synthetic chemists to explore different aspects of the MBH reaction [46,47,48,49,50]. As for the development of new reaction media [51,52,53,54], Basavaiah and Reddy have already demonstrated the usefulness of silica gel as a reaction medium for this reaction [51]. Therefore, we simply describe here a few additional results previously obtained by us [55].

As expected from its high nucleophilicity, 1,4-diazabicyclo[2.2.2]octane (DABCO) was proved to be the most effective catalyst among other amines examined like 4-dimethylaminopyridine (DMAP), triethylamine, and DBU, and as shown in Table 7, alumina, molecular sieves (MS4A), and NH-silica were found to be less effective than silica gel (entries 1-3 *vs.* 4). The reaction rate notably decreased when wet silica gel was employed (entry 5). Thus, the use of dried silica gel in combination with 1.5 eq of DABCO effectively promoted the reaction at room temperature affording the desired products in good isolated yields in a significantly short period of time for this type of reaction (entries 6–8).

**Table 7:** The Morita-Baylis-Hillman reaction in various solid media^ a^. 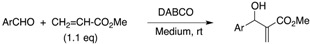

Entry	ArCHO	DABCO (eq)	Medium	Time (h)	Yield (%) ^b^
1	4-NO_2_C_6_H_4_CHO	1.1	alumina	5	62
2	4-NO_2_C_6_H_4_CHO	1.1	MS4A	5	51
3	4-NO_2_C_6_H_4_CHO	1.1	NH-silica ^c^	5	42
4^ d^	4-NO_2_C_6_H_4_CHO	1.1	silica gel	4	83
5^ e^	4-NO_2_C_6_H_4_CHO	1.1	silica gel	15	79
6	4-NO_2_C_6_H_4_CHO	1.5	silica gel	4	90 ^f^
7	4-F-3-NO_2_C_6_H_3_CHO	1.5	silica gel	3.5	77 ^f^
8	C_6_F_5_CHO	1.5	silica gel	3.5	79 ^f^

^a^ An aldehyde (0.5 mmol), methyl acrylate (5.5 mmol), and the medium (500 mg) were used; ^b^ GC yield using *n*-dodecane as a standard, unless otherwise stated; ^c^ Cromatorex^®^ NH-DM1020 (75–150 μm, aminopropyl-modified type, Fuji Silysia Chemical); ^d^ Methyl acrylate (1.2 eq) was used; ^e^ Water (0.1 eq) was used as an additive; ^f^ Isolated yield.

## 3. Experimental

### 3.1. General

Infrared (IR) spectra were recorded on a Shimadzu FTIR-8600 spectrometer and JASCO FT/IR-4200. ^1^H-NMR and ^13^C-NMR spectra were measured on JEOL JNM-EX 400 and Bruker ADVACE III 600. Chemical shifts are given by δ relative to that of internal Me_4_Si (TMS) or the solvent (chloroform-*d* at 77.0 ppm in ^13^C-NMR). Mass spectra were obtained with Shimadzu GC-MS QP-5000. Fast atom bombardment mass spectra (FAB-MS) were obtained with Shimadzu/Kratos CONCEPT 1S or JEOL JMS-DX 303. Elemental analyses were performed at the service center of the elementary analysis of organic compounds, Kyushu University. High-resolution mass spectra (HRMS) were obtained on JEOL JMS-HX100A. Analytical thin layer chromatography (TLC) was performed on a silica gel plate (Merck, Silica gel 60 F_254_, 20 × 20 cm, 0.25 mm). Column chromatography was carried out with silica gel [Silica gel 60 (63–210 μm, Merck), or Silica gel 60N (63–210 μm, Kanto Chemical)] as an adsorbent. In experiments that required solvents and ethylbenzene were purchased from Sigma-Aldrich in an “anhydrous” form and used without any purification. Silica gel 40 (0.2–0.5 mm, Merck), Silica gel 60 (0.2–0.5 mm, Merck), BW-300 (40 μm, Fuji Silysia), Silica gel 60 (40–50 μm, Kanto Chemical), Silica gel 60 N (63–210 μm, Kanto Chemical), COSMOSIL 75SL-II-PREP (42–105 μm, Nacalai Tesque) and Cromatorex^®^ NH-DM1020 (75–150 μm, aminopropyl-modified type, Fuji Silysia Chemical) were examined as the reaction medium. All reactions were carried out under argon. Other commercially available compounds were purchased from Tokyo Chemical Industry Co., Ltd., Wako Pure Chemical Industries, Ltd., Kanto Chemical Co., Inc., Nacalai Tesque Inc. and Sigma-Aldrich Co., and used without further purification. New products were fully characterized after purification by their physical constants, spectral and elemental analyses. For the products that are commercially available or already known compounds, the NMR and MS (in part) data as well as the CAS-registry numbers are given.

### 3.2. General Procedure for the Nitration of Aromatic Compounds on Silica Gel

A typical procedure is given for the preparation of nitronaphthalene. Pre-dried (at 110 °C for 8 h in vacuo) and stocked silica gel [COSMOSIL 75SL-II-PREP (Nacalai Tesque), 2.5 g] was charged in a round-bottom flask and dried for 5 min by heat gun (ca. 300 °C) in *vacuo* just before use. Naphthalene (1.28 g, 10 mmol) was added to the flask, and the mixture was stirred for 30 min at ambient temperature (*ca.* 25 °C). An aqueous 69% HNO_3_ solution (*d *= 1.42, 1.27 mL, 20 mmol) was gradually injected into the mixture over 1 h by syringe pump, and the mixture was stirred for 12 h. The reaction mixture was moved into a short column and eluted with ether. The eluate was washed with water, saturated NaHCO_3_ and brine, and dried over Na_2_SO_4_. After evaporation of the solvent, the residue was purified by column chromatography on silica gel (*n*-hexane/EtOAc = 19/1) to give 1.67 g (97%) of nitronaphthalene as a mixture of isomers (1-nitro/2-nitro = 96.5:3.5), recrystallization of which from ethanol gave pure 1-nitronaphthalene (1.4 g, 81%).

*Ethylnitrobenzene (o-, p-isomer mixture)* [29]. A colorless oil; ^1^H-NMR (CDCl_3_) δ 8.15 (d, 2H, *J* = 8.5 Hz, *p*-isomer), 7.87 (d, 1H, *J* = 8.0 Hz, *o*-isomer), 7.53 (t, 1H, *J* = 8.0 Hz, *o*-isomer), 7.38–7.31 (m, 2H+2H, mixture of isomers), 2.92 (q, 2H, *J* = 7.5 Hz, *o*-isomer), 2.76 (q, 2H, *J* = 7.6 Hz, *p*-isomer), 1.29 (t, 3H, *J* = 7.5 Hz, *o*-isomer), 1.28 (t, 3H, *J* = 7.6 Hz, *p*-isomer); ^13^C-NMR (CDCl_3_) δ 149.1, 138.7, 132.8, 131.0, 126.6, 124.3, 25.9, 14.7 (o-isomer), 151.9, 146.0, 128.5, 123.4, 28.7, 14.8 *p*-isomer); CA Registry No. 612-22-6 (*o*-isomer), 100-12-9 (*p*-isomer).

*2-Nitro-m-cresol* [23]. Yellow solid; ^1^H-NMR (CDCl_3_) δ 10.32 (s, 1H), 7.37 (dd, *J* = 8.4, 7.5 Hz, 1H), 7.01 (ddq, *J* = 8.4, 1.5, 0.6 Hz, 1H), 6.83 (ddq, *J* = 8.4, 1.5, 0.6 Hz, 1H), 2.62 (s, 3H); ^13^C-NMR (CDCl_3_) δ 155.3, 136.8, 135.3, 135.2, 124.0, 117.6, 22.4; CA Registry No. 4920-77-8.

*4-Nitro-m-cresol* [23]. Yellow solid; ^1^H-NMR (CDCl_3_) δ 8.06–8.04 (m, 1H), 6.77–6.75 (m, 2H), 5.96 (s, 1H), 2.61 (s, 3H); ^13^C-NMR (CDCl_3_) δ 159.8, 142.2, 137.5, 127.9, 118.9, 113.6, 21.5; CA Registry No. 2581-34-2.

*6-Nitro-m-cresol* [23]. Yellow solid; ^1^H-NMR (CDCl_3_) δ 10.61 (s, 1H), 7.98 (d, 1H,*J* = 8.7 Hz), 6.94 (ddq, 1H, *J* = 1.9, 0.8, 0.4 Hz), 6.78 (ddq, 1H, *J* = 8.7, 1.9, 0.6 Hz), 2.40 (s, 3H); ^13^C-NMR (CDCl_3_) δ 155.1, 149.8, 131.7, 124.9, 121,6, 119.6, 21.9; CA Registry No. 700-38-9.

*Methyl-p-benzoquinone* [16]. Yellow solid; ^1^H-NMR (CDCl_3_) δ 6.77 (d, 1H, *J* = 10.0 Hz), 6.72 (dd, 1H, *J* = 10.0, 2.5 Hz), 6.62 (dq, 1H, *J* = 2.5, 1.7 Hz), 2.07 (d, 3H, *J* = 1.7 Hz); ^13^C-NMR (CDCl_3_) δ 187.7, 187.6, 145.9, 136.6, 136.5, 133.3, 15.8; CA Registry No. 553-97-9.

### 3.3. General Procedure for the Silica Gel-Mediated One-Pot Wittig Olefination of Aldehydes

Typical procedure is given for the preparation of ethyl cinnamate: To silica gel (Merck’s Silica gel 40, 1 g) were added successively benzaldehyde (104.8 µL, 1 mmol), ethyl chloroacetate (108 µL, 1 mmol), diisopropylethylamine (175.1 µL, 1 mmol), and triphenylphosphine (265 mg, 1 mmol) [or tri-*n*-butylphosphine (202 mg, 1 mmol)] and the whole mixture was stirred for 6 h at 90 °C (or for 2 h at room temperature). The reaction mixture was moved into a short column and eluted with ether. The eluate was concentrated and purified by preparative TLC on silica gel to give 176.1 mg (>99%, *E/Z *= 93/7) of ethyl cinnamate.

*Ethyl Cinnamate* [56]*. * An oil; ^1^H-NMR (CDCl_3_) δ 7.69 (d, 1H, *J* = 16.1 Hz), 7.51–7.54 (m, 2H), 7.37–7.40 (m, 3H), 6.44 (d, 1H, *J* = 16.1 Hz), 4.27 (dd, 2H, *J* = 14.2, 7.3 Hz), 1.34 (t, 3H, *J* = 7.3 Hz); CA Registry Nos. 4192-77-2 (*E*-isomer), 4610-69-9 (*Z*-isomer).

*Ethyl 4-Methoxycinnamate* [57]*. *An oil (96%, *E/Z* = 94/6); ^1^H-NMR (CDCl_3_) δ 7.64 (d, 1H, *J* = 16.1 Hz), 7.48 (dd, 2H, *J* = 6.8, 2.0 Hz), 6.90 (dd, 2H, *J* = 6.8, 2.0 Hz), 6.31 (d, 1H, *J* = 16.1 Hz), 4.25 (dd, 2H, *J* = 14.2, 7.3 Hz), 3.84 (s, 3H), 1.33 (t, 3H, *J* = 7.3 Hz); CA Registry Nos. 24393-56-4 (*E*-isomer), 51507-22-3 (*Z*-isomer).

*Ethyl 4-Methylcinnamate * [57]. An oil (93%, *E/Z *= 94/6); ^1^H-NMR (CDCl_3_) δ 7.66 (d, 1H, *J* = 16.1 Hz), 7.42 (d, 2H, *J* = 8.3 Hz), 7.19 (d, 2H, *J* = 8.3 Hz), 6.39 (d, 1H, *J* = 16.1 Hz), 4.26 (dd, 2H, *J* = 14.2, 7.3 Hz), 2.37 (s, 3H), 1.34 (t, 3H, *J* = 7.3 Hz); CA Registry Nos. 24393-49-5 (*E*-isomer), 97585-04-1 (*Z*-isomer).

*Ethyl 4-Chlorocinnamate* [56]. An oil (99%, *E/Z *= 92/8); ^1^H-NMR (CDCl_3_) δ 7.63 (d, 1H, *J* = 16.1 Hz), 7.45 (dd, 2H, *J* = 6.8, 2.0 Hz), 7.36 (dd, 2H, *J* = 6.8, 2.0 Hz), 6.41 (d, 1H, *J* = 16.1 Hz), 4.27 (dd, 2H, *J* = 14.2, 7.3 Hz), 1.34 (t, 3H, *J* = 7.3 Hz); CA Registry Nos. 24393-52-0 (*E*-isomer), 63757-30-2 (*Z*-isomer).

*Ethyl 4-Cyanocinnamate* [56]. Colorless needles (96%, *E/Z *= 90/10); ^1^H-NMR (CDCl_3_) δ 7.68 (d, 2H, *J* = 8.3 Hz), 7.66 (d, 1H, *J* = 16.1 Hz), 7.61 (d, 2H, *J* = 8.3 Hz), 6.52 (d, 1H, *J* = 16.1 Hz), 4.29 (dd, 2H, *J* = 14.4, 7.1 Hz), 1.35 (t, 3H, *J* = 7.1 Hz); CA Registry Nos. 62174-99-6 (*E*-isomer), 92636-30-1 (*Z*-isomer).

*Ethyl 4-Nitrocinnamate* [56]. Light yellow needles (83%, *E/Z *= 90/10); ^1^H-NMR (CDCl_3_) δ 8.25 (d, 2H, *J* = 8.8 Hz), 7.71 (d, 1H, *J* = 16.1 Hz), 7.67 (d, 2H, *J* = 8.8 Hz), 6.56 (d, 1H, *J* = 16.1 Hz), 4.30 (dd, 2H, *J* = 14.2, 7.3 Hz), 1.36 (t, 3H, *J* = 7.3 Hz); CA Registry Nos. 24393-61-1 (*E*-isomer), 51507-21-2 (*Z*-isomer).

*Ethyl 2-Nonenoate* [43]. An oil (54%, *E/Z *= 85/15); ^1^H-NMR (CDCl_3_) δ 6.97 (dt, 1H, *J* = 15.6, 7.3 Hz), 5.81 (dt, 1H, *J* = 15.6, 1.5 Hz), 4.18 (dd, 2H, *J* = 14.2, 7.3 Hz), 2.19 (dd dd, 2H, *J* = 14.6, 8.8, 7.3, 1.5 Hz), 1.44 (dd, 2H, *J* = 14.6, 7.3 Hz), 1.24–1.35 (m, 6H), 1.29 (t, 3H, *J* = 7.3 Hz), 0.88 (t, 3H, *J* = 6.8 Hz); CA Registry Nos. 38112-59-3 (*E*-isomer), 72284-17-4 (*Z*-isomer).

*Ethyl 3-Cyclohexylacrylate* [57]. An oil (99%, *E/Z *= 97/3); ^1^H-NMR (CDCl_3_) δ 6.91 (dd, 1H, *J* = 16.1, 6.8 Hz), 5.76 (dd, 1H, *J* = 16.1, 1.5 Hz), 4.18 (dd, 2H, *J* = 14.2, 7.3 Hz), 2.13 (dt, 1H, *J* = 6.8, 1.5 Hz), 1.66–1.78 (m, 4H), 1.31–1.08 (m, 9H); CA Registry Nos. 17343-88-3 (*E*-isomer), 18521-02-3 (*Z*-isomer).

*Ethyl 5-Phenyl-2-pentenoate* [56]. An oil (46%, *E/Z *= 87/13); ^1^H-NMR (CDCl_3_) δ 7.29 (t, 2H, *J* = 7.3 Hz), 7.17–7.22 (m, 3H), 7.00 (dt, 1H, *J* = 15.6, 6.8 Hz), 5.85 (dt, 1H, *J *= 15.6, 1.5 Hz), 4.18 (dd, 2H, *J* = 14.2, 7.3 Hz), 2.78 (t, 2H, *J* = 7.3 Hz), 2.52 (dd, 2H, *J* = 7.3, 1.5 Hz), 1.28 (t, 3H, *J* = 7.3 Hz); CA Registry Nos. 55282-95-6 (*E*-isomer), 88842-13-1 (*Z*-isomer).

*Stilbene*[58]. Colorless solid (66%, *E/Z *= 67/33); ^1^H-NMR (CDCl_3_) δ 7.17–7.38 (m, 10H, *E*-isomer), 7.13–7.27 (m, 10H, *Z*-isomer), 6.61 (s, 2H, *Z*-isomer), 6.60 (s, 2H, *E*-isomer); CA Registry Nos. 103-30-0 (*E*-isomer), 645-49-8 (*Z*-isomer).

*α-Benzylidene-γ-butyrolactone* [59]. Yellow solid (52%, *E/Z *= 95/5); ^1^H-NMR (CDCl_3_) δ 7.59 (t, 1H, *J* = 2.9 Hz), 7.51 (d, 2H, *J* = 6.8 Hz), 7.41–7.47 (m, 3H), 4.48 (t, 2H, *J* = 7.3 Hz), 3.27 (dt, 1H, *J* = 11.7, 2.9 Hz); CA Registry Nos. 30959-91-2 (*E*-isomer), 40011-26-5 (*Z*-isomer).

*Chalcone* [59]. Colorless solid (85%, *E/Z *= 99.7/0.3); ^1^H-NMR (CDCl_3_) δ 7.95–8.04 (m, 2H), 7.82 (d, 1H, *J* = 16.1 Hz), 7.42–7.66 (m, 11H); CA Registry Nos. 614-47-1 (*E*-isomer), 614-46-0 (*Z*-isomer).

### 3.4. General Procedure for the Silica Gel-Mediated Morita-Baylis-Hillman Reaction

Typical procedure is given for the preparation of methyl 2-[hydroxy(4-nitrophenyl)methyl]acrylate: To a mixture of DABCO (0.084 g, 0.75 mmol) and silica gel (Merck’s Silica gel 40, 0.5 g), *p*-nitrobenzaldehyde (0.079 g, 0.5 mmol) and methyl acrylate (0.05 mL, 0.55 mmol) were added. The whole mixture was stirred at room temperature for 4 h. On completion of the reaction, the reaction mixture was moved into a short column and eluted with CH_2_Cl_2_. Evaporation of the solvent afforded the desired product, which was further purified by silica gel chromatography to give the pure product as an oil (0.111 g, 90%).

*Methyl 2-(Hydroxy(4-nitrophenyl)methyl)acrylate* [54]. An oil; ^1^H-NMR (CDCl_3_) δ 8.20 (dt, 2H, *J* = 9.1, 2.1 Hz), 7.57 (dt, 2H, *J* = 9.1, 2.1 Hz), 6.40 (d, 1H, *J* = 0.5 Hz), 5.88 (d, 1H, *J* = 0.5 Hz), 5.64 (d, 1H, *J* = 5.5 Hz), 3.74 (s, 3H), 3.36 (d, 1H, *J* = 6.0 Hz); ^13^C-NMR (CDCl_3_) δ 166.3, 148.9, 147.3, 141.1, 127.4, 127.1, 123.5, 72.3, 52.1; CA Registry No. 114106-93-3.

*Methyl 2-[Hydroxy(4-fluoro-3-nitrophenyl)methyl]acrylate *. A yellow oil (77%); IR (KBr) 3484, 1715, 1540, 1440, 1351, 1154; ^1^H-NMR (CDCl_3_) δ 8.07 (m, 1H), 7.68 (m, 1H), 7.27 (m, 1H), 6.40 (s, 1H), 5.95 (s, 1H), 5.59 (s, 1H), 3.75 (s, 3H), 3.59 (bs, 1H); ^13^C-NMR (CDCl_3_) δ 166.1, 156.03, 140.8, 138.7 (d, *J* = 5.0 Hz), 133.6 (d, *J* = 9.0 Hz), 127.0, 127.0, 124.0 (d, *J* = 2.0 Hz), 118.2 (d, *J* = 21.0 Hz), 71.66, 52.1; HRMS (FAB+) *m/z* calcd for C_11_H_11_O_5_NF (M+H) 256.0621, found 256.0619; Anal. calcd for C_11_H_10_O_5_NF: C: 51.77%; H: 3.95%; N: 5.49%; found: C: 51.71%; H: 3.93%; N: 5.44%.

*Methyl 2-[Hydroxy(2,3,4,5,6-pentafluorophenyl)methyl]acrylate *. Colorless solid (79%); IR (KBr) 3470, 1709, 1524, 1505, 1306, 1063, 997; ^1^H-NMR (CDCl_3_) δ 6.48 (s, 1H), 6.10 (s, 1H), 5.89 (s, 1H), 3.74 (s, 3H), 3.43 (bs, 1H); ^13^C-NMR (CDCl_3_) δ 165.8, 146.3–136.2 (m), 126.6, 114.9 (m), 64.1, 52.0; HRMS (FAB+) *m/z* calcd for C_11_H_8_O_3_F_5_ (M+H) 283.0394, found 283.0395; CA Registry No. 1019127-87-7.

## 4. Conclusions

We have demonstrated the utility of silica gel as a solid reaction medium for some useful organic transformations, in which silica gel served as a drying agent as well as an efficient dispersant providing better yields of the products than those obtained in the corresponding reactions performed in organic solvents or under neat conditions. They are: (1) aromatic nitration using commercial 69% nitric acid at room temperature, (2) one-pot Wittig-type olefination of aldehydes with organic halides by the aid of a phosphine and a base, and (3) the Morita-Baylis-Hillman reaction of aldehydes with methyl acrylate at room temperature. These protocols are highly convenient and environmentally friendly as the reactions proceed under organic solvent-free heterogeneous conditions and, in most cases, simple washing of the reaction mixture-containing silica gel with a minimally required amount of appropriate solvent with low polarity is enough to get the desired products with appreciable purities.
